# IFN-λ4 genetic variants influence clinical malaria episodes in a cohort of Kenyan children

**DOI:** 10.1186/s12936-021-03689-z

**Published:** 2021-04-21

**Authors:** Gabriela Samayoa-Reyes, Conner Jackson, Sidney Ogolla, Katherine Sabourin, Adeola Obajemu, Arlene E. Dent, Ludmilla Prokunina-Olsson, Rosemary Rochford

**Affiliations:** 1 University of Colorado Anschutz Medical Campus, Aurora, CO USA; 2 Center for Global Health Research, Kenya Medical Research Institute, Kisumu, Kenya; 3 Laboratory of Translational Genomics, Division of Cancer Epidemiology and Genetics, National Cancer Institute, National Institutes of Health, Bethesda, MD USA; 4 Center for Global Health and Diseases, Case Western Reserve University, Cleveland, OH USA

**Keywords:** *Plasmodium falciparum*, Malaria, Innate Immunity, Type III Interferon, IFN-λ4

## Abstract

**Background:**

Interferon (IFN)- λ4, a type III IFN, production is controlled by a dinucleotide frameshift variant (rs368234815-dG/TT) within the first exon of the *IFNL4* gene. Carriers of the *IFNL4*-dG allele but not the *IFNL4*-TT allele are able to produce the IFN-λ4 protein. Patients with hepatitis C virus that do not produce the IFN-λ4 protein have higher rates of viral clearance suggesting a potential inhibitory role of IFN-λ4 in liver-tropic infections.

**Methods:**

In this study, it was investigated whether children infected with *Plasmodium falciparum,* which has a well-characterized liver stage infection, would be more susceptible to clinical malaria relative to their *IFNL4*-rs368234815 allele. A cohort of 122 children from a malaria holoendemic region of Kenya was analysed. Episodes of clinical malaria and upper respiratory tract infections (URTIs) were determined using information collected from birth to 2 years of age. The dinucleotide frameshift variant *IFNL4*-rs368234815-dG/TT was genotyped using a TaqMan assay.

**Results:**

In this cohort, 33% of the study participants had the dG/dG genotype, 45% had the dG/TT genotype, and 22% had TT/TT genotype. The number and time to first episode of clinical malaria and URTIs with respect to the *IFNL4*-rs368234815 allele was evaluated. It was found that children that carried the *IFNL4-*rs368234815-dG allele had an increased number of clinical malaria episodes. In addition, there was a significant association between earlier age of first malaria infection with carriers of the *IFNL4*-dG allele (*p-value:* 0.021).

**Conclusion:**

The results suggest that the ability to produce IFN-λ4 negatively affects host immune protection against *P. falciparum* malaria in Kenyan children.

**Supplementary Information:**

The online version contains supplementary material available at 10.1186/s12936-021-03689-z.

## Background

Malaria remains a major global health burden. In 2019, there were 409,000 deaths due to malaria and an estimated 229 million cases worldwide, placing roughly half of the world’s population at risk of infection [1]. The infection is caused by intracellular protozoan parasite of the genus *Plasmodium*, with the species *Plasmodium falciparum* contributing the greatest morbidity and mortality. Transmission occurs through the bite of an infected *Anopheles* mosquito. Sporozoites injected by the mosquito travel to and infect hepatocytes, where they develop to form thousands of merozoites that then infect erythrocytes, resulting in the clinical presentation of the disease.

Type III interferons (IFNs) are antiviral cytokines with a broad antiviral activity that induce hundreds of interferon-stimulated genes (ISGs) [2, 3]. They provide a localized immune response at epithelial surfaces and if this response is successful, type I and type II IFN responses are suppressed [2]. There are four type III IFNs; IFN-λ1, IFN-λ2, IFN-λ3, and the most recently discovered IFN-λ4 [3, 4]. A dinucleotide frameshift variant (rs368234815-dG/TT) within the first exon of the *IFNL4* gene controls the production of IFN-λ4 protein. Roughly half of the world population are carriers of the *IFNL4*-dG allele and are able to produce the IFN-λ4 protein, whereas carriers *IFNL4*-TT allele cannot [4, 5]. The TT allele frequency is at its lowest in Africa (29%) and reaches near-fixation frequency in East Asia (94%) [6]. This is consistent with a population-specific natural selection, in which the TT allele appears beneficial outside of Africa [6]. The reason why the ancestral allele, *IFNL4*-dG, is still retained at high frequencies in the African populations remains unclear.

The effect of the *IFNL4*-rs368234815 frameshift variation has been studied in individuals infected with hepatitis C virus (HCV) [4]. Surprisingly, the *IFNL4*-dG allele (IFN-λ4 is produced) is associated with unfavorable clinical outcomes and the *IFNL4*-TT/TT genotype (IFN-λ4 null) with higher rates of viral clearance; the mechanism behind this paradoxical finding remains unknown [4, 7–9]. One possibility is that IFN-λ4 negatively regulates the Type I IFN responses critical for viral clearance [10]. More recently, it has also been reported that the *IFNL4*-dG allele is associated with reduced clearance of RNA viruses that cause respiratory infections [11]. These studies suggest that carriers of the *IFNL4*-dG allele have different disease risks compared to carriers of the *IFNL4*-TT/TT genotype. Because HCV is a hepatropic virus, it was reasoned that carriers of the *IFNL4*-dG allele might also have differential risk of malaria infection where infection of hepatocytes occurs during the primary stage of infection. In this study, the association between IFN-λ4 polymorphism and risk of clinical malaria and upper respiratory tract infections (URTIs) was investigated in a prospective birth cohort of Kenyan children.

## Methods

### Study design

Samples and clinical data collected by the Chulaimbo Antenatal Postnatal (CHAP) study, a prospective cohort study conducted in Kisumu, Kenya between 2011 and 2015 were used. Details of this cohort have been described elsewhere [12, 13]. Briefly, the study enrolled pregnant women aged 18–45 years presenting for antenatal consultation at Chulaimbo County Hospital (CCH). The eligibility criteria included HIV negative, singleton pregnancy, residency within ten kilometers of the hospital to facilitate follow-up. Mothers were monitored for malaria in pregnancy as described [13], malaria infection at any of the ANC visits or at delivery was considered as positive for maternal malaria. If the participating women gave birth at CCH, newborn children were enrolled at delivery and underwent a newborn exam that included laboratory testing, a physical exam and anthropometric measurements. Children were followed for up to 2 years. The protocol and study procedures were approved by the institutional review board of the SUNY Upstate Medical University (where the study was initiated), COMIRB at University of Colorado, and the Scientific and ethical review unit (SERU) at KEMRI.

The clinical data was collected by completing a structured questionnaire each time the child came for a clinic visit; children then underwent a physical evaluation and any medical findings were included in these questionnaires. URTIs were diagnosed by physical exam by the study clinical officer. Only cases where a diagnosis of URTI were included in the analysis. A small number of cases of pneumonia (< 3%) were identified and these were not included in the URTI definition. Clinical malaria episodes were diagnosed by clinical presentation and confirmed by thick and thin blood smears stained with Giemsa and visualized by microscopy to detect *P. falciparum* parasites in the blood. Finger-prick blood samples collected in EDTA at ~ 6 months of age were stored at −80 °C prior to shipment of samples to the USA.

### Genotyping

Genomic DNA was extracted from whole blood using DNeasy Blood & Tissue Kit (Qiagen) and genotyped for *IFNL4*-rs368234815 by custom TaqMan genotyping assays, using Genotype Master Mix (Qiagen), on BioRad iQ5, with standard conditions as previously described [4]. Testing was performed at University of Colorado by the Rochford laboratory and all testing was blinded to clinical phenotypes.

Glucose-6-Phosphate dehydrogenase deficiency (G6PDd) was characterized as previously published [14]. Briefly, two PCR products, 352 bp and 295 bp, within the *G6PD* gene were amplified by PCR using the following primers: A- Forward (5′-CAGCCACTTCTAACCACACACCT-3′), A- Reverse (5′-CCGAAGCTGGCCATGCTGGG-3′), A + Forward (5′-CTGTCTGTGTGTCTGTCTGTCC-3′) and A + Reverse (5′-GGCCAGCCTGGCAGGCGGGAAGG-3′). The PCR amplicons were subsequently subjected to restriction enzyme digestion using NlaIII with resulting fragment sizes visualized by horizontal gel electrophoresis. For A- an uncut product was found from the normal locus, whereas two DNA fragments, 218- and 134-bp, were generated in the mutant locus. For A + 2 DNA fragments, 243- and 52-bp, were found to be associated with the normal locus and 3 DNA fragments, 141-, 102-, 52-bp, were generated for the mutant locus.

Hb-A/S trait was characterized as previously published [15]. Briefly, a 772-bp PCR product within the human beta-globin gene was amplified from DNA extracted from whole blood using the following primers: HbB1 (5′- TCCTAAGCCAGTGCCAGAAG -3′) and HbB2 (5′-GAATTCGTCTGTTTCCCATTCTAAAC -3′). The PCR amplicon was subsequently subjected to restriction enzyme digestion using Bsu361 with resulting fragment sizes visualized by horizontal gel electrophoresis. A 430-bp DNA fragment was found to be associated with the mutant locus, whereas 228- and 202-bp DNA fragments were generated from the normal locus.

### Statistical analysis

Using data from questionnaires collected on all clinic visits, the relationship between malaria episodes and upper respiratory tract infections (URTI) with respect to *IFNL4* alleles was evaluated. Only malaria episodes or URTIs that were reported on clinic visits forms were counted for. A Negative binomial regression model with an offset for the total number of sick and follow-up visits was used to evaluate the relationship between *IFNL4* alleles and the number of infections; malaria episodes and URTIs were modeled separately. Estimates from the negative binomial model were exponentiated and reported as rate ratios. To evaluate the time to first malaria infection, a Cox proportional hazards model was fit using the survival package (v 3.1–8) [16, 17] and survival plots were created using the survminer package (v 0.4.6) [18] in R. The final Cox model included adjustments for *G6PDd* and sickle cell trait with right-censoring at the end of the 2-year study; the proportional hazards assumption was checked and was not violated. Adjustments for gravidity and maternal malaria exposure did not improve model fit or change conclusions about *IFNL4* associations, so they were not included in the final cox proportional hazards model. To evaluate time to first URTI, Kaplan Meier estimators were calculated, and a log rank test was used to test for differences between *IFNL4* alleles. Tables were created with the Table 1 package (v 1.2.1) [19]; p-values were determined using Pearson’s Chi-squared tests with continuity correction for categorical variables and unequal variance t-tests for continuous variables (following an assessment of normality). All analyses were completed using R (version *3.6.0*) [20].

**Table 1:** Study population demographics as well as gravidity of the mother

	*IFNL4*-dG allele (dG/dG and dG/TT genotypes combined) N = 95	*IFNL4*-TT/TT genotype N = 27	p-value
Sex			
Female	52 (55%)	14 (52%)	0.963
Male	43 (45%)	13 (48%)	
Birth weight (grams)			
Mean (SD)	3230 (429)	3000 (405)	0.024
Median [Min., Max.]	3200 [2200, 4000]	3000 [2100, 3800]	
Missing	10 (11%)	3 (11%)	
First born			
No	62 (65%)	15 (56%)	0.486
Yes	33 (35%)	12 (44%)	
Number of visits			
Mean (SD)	12.4 (5.6)	12.6 (6.3%)	0.899
Median [Min., Max.]	13.0 [1.0, 29.0]	13.0 [2.0, 25.0]	

Gender, birth weight and median number of visits of the Kenyan children enrolled for the study as well as gravidity of the mother

## Results

### Characteristics of study population

To evaluate whether genetic variants in IFN-λ4 play a role in *P. falciparum* and upper respiratory tract infection frequency, clinical data collected from 122 children that were part of a previously described birth cohort based in Western Kenya where malaria transmission is holoendemic [12, 13] was analysed. The demographics of this study population, including child gender, birth weight and median number of clinical visits that they had during the 2-year follow-up are described in Table 1. In order to further evaluate the observed significant difference in birth weight between *IFNL4*- rs368234815 dG allele (herein *IFNL4*- dG allele) and the *IFNL4*-rs368234815 TT/TT genotype, the relationship between maternal malaria during pregnancy and *IFNL4* was analysed and no significant difference was found between the two groups (Chi-square test; p-value: 0.297). Furthermore, for this population, there was not a significant relationship between birthweight and maternal malaria exposure (Welch t-test; p-value: 0.779).

**Fig. 1:**
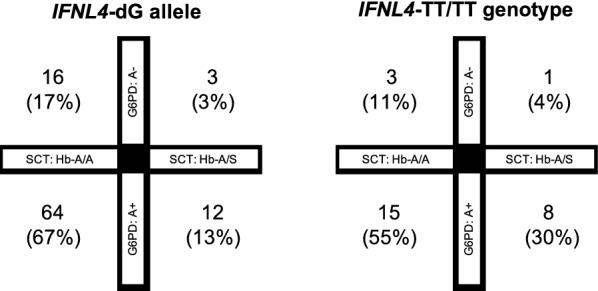
Sickle cell trait genotype and G6PD allelic determinant stratified by I*FNL4* allele. The study subjects were divided into two groups based on presence or absence of an *IFNL4*-dG allele. Within each group, subjects were further stratified based on carriage of sickle cell trait (SCT) and Glucose-6-Phosphate (G6PD) deficiency. Most of the subjects, 67% for the *IFNL4*-dG allele group and 55% for the *IFNL4*-TT/TT genotype group had neither the G6PD deficiency allelic determinant nor were carriers of the sickle cell trait. On the contrary, only 3% of the subjects with the *IFNL4*-dG allele were both carriers of the sickle cell trait and G6PD deficiency allelic determinant, while the percentage of subjects that had both genes in the *IFNL4*-TT/TT genotype group was 1%

Genomic DNA was extracted from blood collected at 6 months of age and was genotyped for the *IFNL4*-rs368234815 polymorphism using a custom TaqMan genotyping assay (Additional file 1: Fig. S1 shows the allelic discrimination plot generated from the genotyping assay [see Additional file 1]). In addition, samples were genotyped for glucose-6-phosphate dehydrogenase deficiency (G6PDd) and sickle cell trait (*HbB*-rs334-T/A); both of which are associated with resistance to blood stage malaria infection [15, 21–24]. The observed *IFNL4*- dG allele frequency in the study population was 78% with a genotype frequency of 33%, 45% and 22% for dG/dG, dG/TT and TT/TT genotypes, respectively. This distribution was consistent with Hardy–Weinberg equilibrium (HWE *p-value:* 0.945), telling us that the genotype frequencies seen are a simple function of allele frequency and will remain constant from one generation to the next in the absence of evolutionary influence or other disturbing factors. In addition, 23 (19%) study participants had G6PD deficiency (*IFNL4*- TT/TT genotype = 19; *IFNL4*-dG allele = 4; Chi-square test; p-value: 0.7432) and 24 (20%) were found to be carriers of the sickle cell trait (Hb-A/S) (*IFNL4*- TT/TT genotype = 15; *IFNL4*-dG allele = 9; Chi-square test; p-value: 0.080) (Table 2). Figure 1 presents a diagram of sickle cell trait genotype and G6PD allelic determinant stratified by *IFNL4* allele.

**Table 2:** *IFNL4* genotype and allelic determinant for G6PD deficiency and SCT genotype; stratified by *IFNL4* allele

	*IFNL4*-dG allele (dG/dG and dG/TT genotypes combined) N = 95 (%)	*IFNL4*-TT/TT genotype (N = 27) (%)	Overall (N = 22) (%)
*IFNL4* genotype			
dG/dG	40 (42)	0 (0)	40 (33)
dG/TT	55 (58)	0 (0)	55 (45)
TT/TT	0 (0)	27 (100%)	27 (22)
G6PD deficiency allelic determinant			
A-(G6PD deficiency)	19 (20)	4 (15)	23 (19)
A + (G6PD wild type)	76 (80)	23 (85)	99 (81)
Sickle cell trait genotype			
Hb-A/A (wild type)	80 (84)	18 (67)	98 (80)
Hb-A/S (sickle cell trait carriers)	15 (16	9 (33)	24 (20)

Genetic distribution of study participants including *IFNL4* genotype, allelic determinant for glucose-6-phosphate (G6PD) deficiency and SCT (sickle cell trait) genotype; all stratified by *IFNL4* allele

### Carriage of the IFNL4-dG allele does not influence frequency of URTIs

To address whether there were effects on malaria or URTI, health records from a total of 1,520 clinic visits occurring between birth to 2 years of age were reviewed for the 122 children. Children that were carriers of the *IFNL4*- dG allele (both dG/dG and dG/TT genotype) were grouped together and compared to those that did not have a dG allele (*IFNL4*-rs368234815 TT/TT genotype- herein *IFNL4*-TT/TT).

**Fig. 2:**
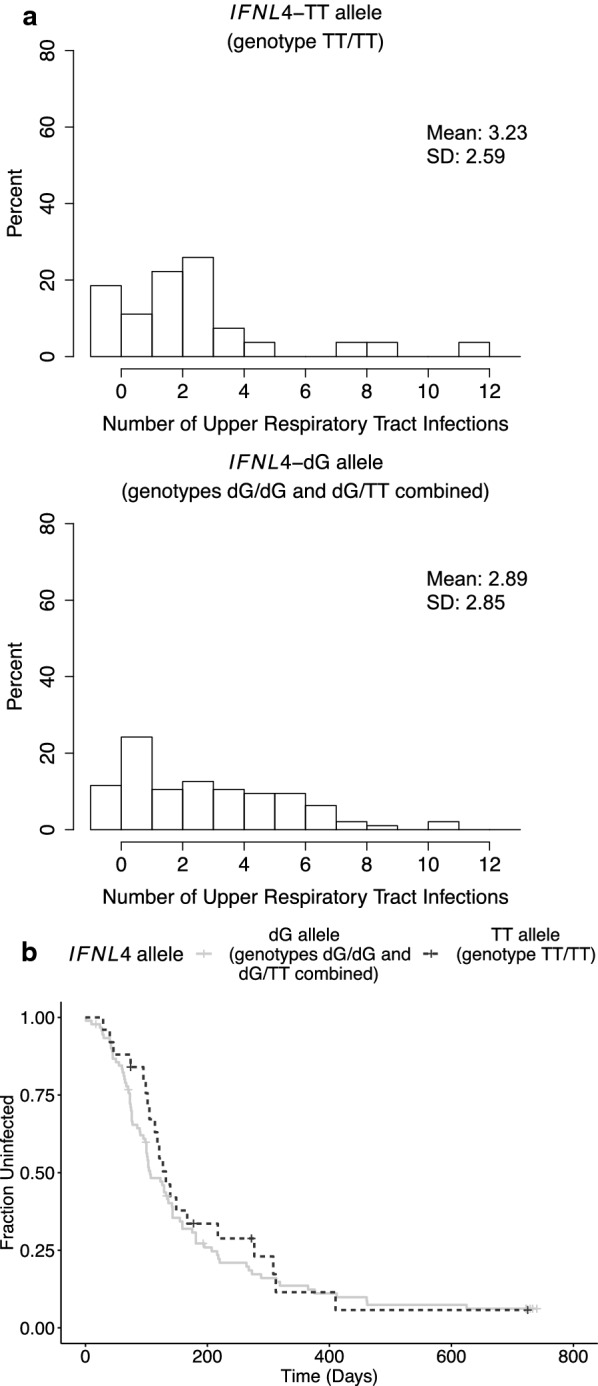
Incidence of URTIs and time to first infection in children from the CHAP prospective cohort study. The study subjects were grouped based on presence of a *IFNL4*-dG allele, dG/dG and dG/TT genotypes (n = 95) and compared with children that did not carry a dG allele, TT/TT genotype (n = 27). **a** Histograms showing the distribution of URTIs in the study population during the first 2 years of life presented based on *IFNL4*-rs368234815 polymorphism. The mean number of URTIs and standard deviation (SD) is included. The rate of URTI’s was 11.80% lower for those that had the IFNL4- TT/TT genotype relative to those that had an IFNL4-dG allele after adjusting for the number of visits (95% CI −32.76%, 14.46%; *p-value: *0.355). **b** Kaplan–Meier survival curve showing time to first URTIs based on *IFNL4*-rs368234815 polymorphism. No significant difference between *IFNL4*-rs368234815 genotype was found (*p-value*:0.512)

A negative binomial regression model was used to examine if there was an association between *IFNL4*-rs368234815 polymorphism and the number of URTIs during the first 2 years of life. It was found that URTI’s rate was 11.80% lower for those that had the *IFNL4*- TT/TT genotype relative to those that had an *IFNL4*-dG allele after adjusting for the number of visits (95% CI −32.76%, 14.46%; *p-value:* 0.355) (Fig. 2a). The association with time to first URTI was analysed, no significant difference between the *IFNL4*- dG allele and the *IFNL4*- TT/TT genotype with time to first URTIs (*p-value:* 0.512) was found (Fig. 2b).

### Carriage of the IFNL4-dG allele does influence age of first malaria infection

**Fig. 3:**
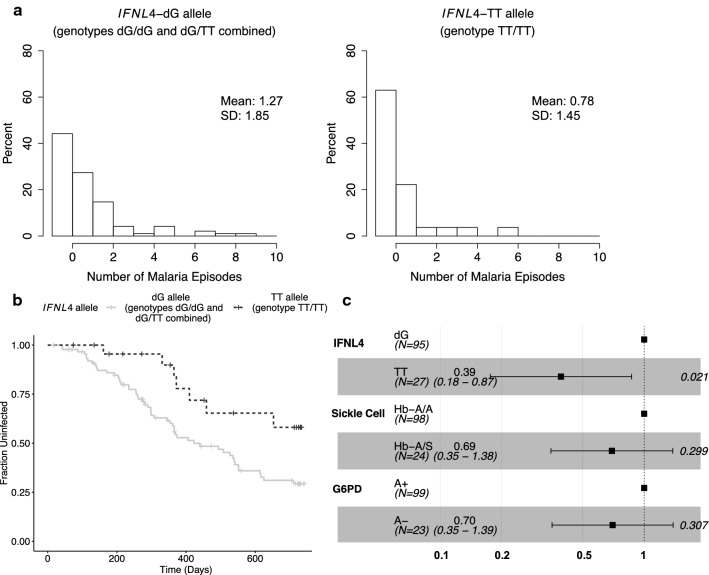
Frequency and time to first Malaria episodes in relation to *IFNL4*-rs368234815 polymorphism. The 122 study subjects were grouped based on presence of a *IFNL4*-dG allele, dG/dG and dG/TT genotypes (n = 95) and compared with children that did not carry a dG allele, TT/TT genotype (n = 27). **a** Histograms showing the distribution of malaria episodes in the study population during the first 2 years of life presented based on *IFNL4*-rs368234815 polymorphism. The mean and standard deviation (SD) of malaria episodes is included. After accounting for the number of visits, cases of malaria were found to be 38.75% lower for those with the *IFNL4*- TT/TT genotype relative to those that had a *IFNL4*-dG allele (95% CI −66.70%, 10.14%; *p-value:* 0.111). **b** Kaplan–Meier survival curve showing time to first malaria episode based on *IFNL4*-rs368234815 polymorphism. Earlier timing of the first malaria infection was associated with *IFNL4*-dG allele (*p-value:* 0.019) as compared to children with the *IFNL4*-TT/TT genotype. **c** Forest plot showing the results of a Cox proportional hazard model that takes into consideration the effect of two genetic traits that are known to be associated with resistance to blood stage malaria infection, G6PDd and sickle cell, on time to first episode. Adjustments for gravidity and maternal malaria exposure did not improve model fit, so they were not included for Cox proportional hazards model. Hazard ratios are reported along with confidence intervals and p-values for *IFNL4* (dG allele, dG/dG and dG/TT genotypes and TT allele, TT/TT genotype), G6PD alleles and sickle cell genotypes. A significant hazard ratio (*p-value:* 0.021) is observed for the *IFNL4* genotype, indicating reduced malaria risk for those with the *IFNL4*-TT/TT genotype

Furthermore, it was also evaluated whether carriage of the *IFNL4*-dG allele was associated with frequency of malaria infections or time to first malaria infection in study participants (Fig. 3), using the same regression framework. After offsetting for the number of visits, cases of malaria were found to be 38.75% lower for those with the *IFNL4*- TT/TT genotype relative to those that had a *IFNL4*-dG allele (95% CI −66.70%, 10.14%; *p-value:* 0.111) (Fig. 3a). Although the results are not significant, the trend suggests that those with the *IFNL4*- TT/TT genotype potentially have fewer cases of malaria. Next the association between *IFNL4*-rs368234815 polymorphism and time to first malaria episode was analysed. It was found that children with an *IFNL4*-dG allele were more likely to have an earlier occurrence of the first malaria infection compared to children with the *IFNL4*- TT/TT genotype (log-rank *p-value:* 0.019) (Fig. 3b). Children carrying the *IFNL4*-TT/TT genotype had a reduced hazard of earlier episodes of malaria compared to children with the *IFNL4*-dG allele (unadjusted hazard ratio: 0.38, 95% CI 0.17, 0.83; *p-value*: 0.016). For this population, there was not a statistically significant relationship between either G6PDd or sickle cell trait and time to first malaria infection, although there was a trend towards reduced hazard of earlier episodes of first malaria (G6PDd (A-) unadjusted hazard ratio: 0.62, 95% CI 0.31, 1.23; *p-value*: 0.173 and sickle cell trait (Hb-A/S allele) unadjusted hazard ratio: 0.77, 95% CI 0.39, 1.52; *p-value*: 0.307). Because of the known association of G6PDd and sickle cell trait with protection from clinical malaria, a multivariable Cox proportional hazards model to control for the G6PDd and sickle cell trait was performed. There was no difference in the direction, magnitude, or statistical conclusion following adjustment (Fig. 3c).

## Discussion

Malaria remains a major burden worldwide but more so in the African population which has the highest malaria associated morbidity and mortality especially in children [1]. A number of studies have explored the driving force of malaria on human evolution with a specific focus on immune alleles [25]. This study evaluated whether the ancestral *IFNL4*- dG allele was protective against *P. falciparum* malaria in an infant cohort in Kisumu, Kenya a malaria-endemic region with a high rate of transmission. It was found that carriage of the *IFNL4*- dG allele (IFN-λ4 is produced), increased the number of clinical malaria episodes and was associated with an earlier time to first malaria infection during the first 2 years of life as compared to children with the *IFNL4*- TT/TT genotype. These suggests that the ability to produce IFN-λ4 negatively affects the ability to protect against *P. falciparum* in Kenyan children.

The results presented here are consistent with several studies that have looked at the relationship between carriers of the *IFNL4*- dG allele and HCV infection and found that the ability to produce IFN-λ4 (e.g. in carriers of *IFNL4*- dG allele) has a negative effect on viral clearance either spontaneously or after treatment [4, 7–9]. Why expression of IFN-λ4 correlates with increased malaria disease risk in infancy remains unknown. This is counterintuitive to what is known of interferons’ capacity to induce an antiviral state in infected and uninfected cells to block viral replication and spread of infection [2]. One possibility is that IFNs have a different effect on intracellular parasitic infection as compared to viral infections. Alternatively, recent studies have shown that IFN-λ4 acts faster than the other type III IFNs and that its extremely strong antiviral response induces negative regulators, like USP18, to prevent other IFN responses from being mounted [26]. Based on these it is likely that in *IFNL4*- dG allele carriers, IFN-λ4 is the first response mounted against *P. falciparum* in the liver but this response alone cannot clear infection and other IFN responses are inhibited. It was recently shown in a rodent malaria model using *Plasmodium berghei,* that liver stage infection is sensed by the host and activates a type I IFN response that is able to control parasite load and mediate host resistance to reinfection [27]. This led us to hypothesize that in *IFNL4*- dG allele carriers (produce IFN-λ4) type I IFN response would be suppressed, via the negative regulators induced by IFN-λ4, and the host would not be able to control parasite load, leading to a higher number of malaria episodes.

Type III IFNs are known to be the first line of defense for respiratory RNA viruses [28]. For example, a recent study conducted with children from Rwanda showed a reduced clearance of RNA viruses that cause respiratory infections in children carrying the *IFNL4*-dG allele [11]. This study found that carriers of the *IFNL4*- dG allele had more URTIs during the first 2 years of life, although this was not statistically significant. These results are consistent with the study in Rwanda and suggests that the capacity to express IFN-λ4 may increase the risk of URTIs.

The reason why the ancestral*, IFNL4-*dG allele, is still conserved in the African population and has not changed to the derived human-specific allele, *IFNL4-*TT allele, remains unknown. Carriage of the *IFNL4-dG* allele is clearly not providing protection against HCV [4, 7–9], respiratory infections [11], gastrointestinal infections [29], human coronavirus, HCoV-229E or MERS-CoV [30], and now this study shows similar results for *P. falciparum* malaria in children. Undoubtedly there are other selection pressures conserving this allele in this population.

Interestingly, in a study using the rodent *Plasmodium* species *Plasmodium yoelii* and infection of mice, it was reported that the absence of IFNλ signaling decreased parasite burden and increased early antibody titers [31]. This suggests that IFNλ receptor expression plays a role in suppressing the humoral immune response to blood-stage malaria and impedes acute parasite clearance during primary blood-stage malaria infection. This led us to ask if the detrimental role that expression of IFNλ4 (carriers of the *IFNL4*-dG allele) has during malaria infection is due to its effect on blood or liver stage of *P. falciparum* infection? It is shown here that after adjusting for G6PD deficiency (carriage of the A^−^ G6PD allele) or sickle cell trait (Hb-A/S allele), both associated with resistance to blood stage malaria infection, carriers of the *IFNL4* TT/TT genotype had a reduced risk for malaria infection. These results would argue that *IFNL4* action is more important for modulating the liver stage of malaria infection as compared to a role in blood stage infection.

A main strength of this study is that it was a prospective cohort study that followed children from birth to 2 years of age and this allowed us to measure malaria episodes over time. A limitation of this study was a small sample size, which in some cases (e.g. URTI’s) hindered the ability to detect a significant difference between carriers of the different *IFNL4* alleles. In addition, the study was not designed to evaluate the cause of URTI’s and, therefore, might have missed disease specific effects. There is also a likelihood that not all children came to the clinic when they were exhibiting symptoms potentially resulting in a failure to capture some episodes of infection. It is important to mention that since these children were part of a cohort study, they had access to regular medical care potentially reducing the frequency of infections and the severity of malaria observed. One of the questions that was not addressed in this 2-year study is if the ability to produce IFN-λ4, which correlates to earlier time to first infection and more clinical malaria episodes during infancy has any long-term detrimental effects on this pediatric study population.

## Conclusion

In conclusion, this study suggests production IFN-λ4 (e.g. carriers of *IFNL4*- dG allele) negatively affects the ability to protect against *P. falciparum* malaria during infancy in children living in a malaria holoendemic region of East Africa. These results are consistent with a longitudinal study of children in Mali, West Africa [29]. The underlying mechanism as to why the ability to express a type III interferon is not protective against a parasitic infection remains an important question for future studies.

It is demonstrated that production of IFN-λ4, a type III interferon (IFN), had a detrimental effect on carriers, increasing the frequency of infections and time to first malaria infection during infancy, presenting a risk for the host. This finding is paradoxical from the known role of interferons in inducing an antiviral state in cells suggesting a different mechanism for function of IFN-λ4 in parasitic infections. Production of IFN-λ4 suppresses the type I IFN response, via the induction of negative regulators. In rodent models, the type I IFN response can control *Plasmodium* parasite load; without type I IFN response due to production of IFN-λ4, the host would not be able to control parasite load potentially leading to a higher number of malaria episodes.

## Supplementary Information

**Additional file 1: Fig. S1.** Representative allelic discrimination plot for genotyping of IFNL4-rs368234815 polymorphism by custom TaqMan genotyping assay. A clear separation of the homozygotes is shown, heterozygotes on the other hand show presence of both alleles, with different expression levels between samples. HapMan controls are shown in duplicate, inside boxes, and five randomly selected study subjects gDNA samples are also included.

## Data Availability

Not applicable.
