# Supplementary material for: IFN-λ4 genetic variants influence clinical malaria episodes in a cohort of Kenyan children
Source: Malar J. 2021 Apr 21;20:196. doi: 10.1186/s12936-021-03689-z (PMC8058600; doi:10.1186/s12936-021-03689-z)
Supplement: Supplementary file 1 — Additional file 1: Fig. S1. Representative allelic discrimination plot for genotyping of IFNL4-rs368234815 polymorphism by custom TaqMan genotyping assay. A clear separation of the homozygotes is shown, heterozygotes on the other hand show presence of both alleles, with different expression levels between samples. HapMan controls are shown in duplicate, inside boxes, and five randomly selected study subjects gDNA samples are also included. [file 12936_2021_3689_MOESM1_ESM.docx]

**IFN-λ4 genetic variants influence clinical malaria episodes in a cohort of Kenyan children**

**Additional file 1**

Gabriela Samayoa-Reyes^1^, Conner Jackson^1^, Sidney Ogolla^2^, Katherine Sabourin^1^, Adeola Obajemu^3^, Arlene E. Dent^4^, Ludmilla Prokunina-Olsson^3^, Rosemary Rochford^1*^

^1^University of Colorado Anschutz Medical Campus, Aurora, CO, United States, ^2^Center for Global Health Research, Kenya Medical Research Institute, Kisumu, Kenya, ^3^Laboratory of Translational Genomics, Division of Cancer Epidemiology and Genetics, National Cancer Institute, National Institutes of Health, Bethesda, MD, United States, ^4^Center for Global Health and Diseases, Case Western Reserve University, Cleveland, OH, United States

* corresponding author

**Email:**  [rosemary.rochford@cuanschutz.edu](mailto:rosemary.rochford@cuanschutz.edu)

**Address:** 12800 East 19^th^ Avenue P18-9403D Aurora, CO 80045

**Phone number:** (303) 724-9960

**Supplementary Figure**


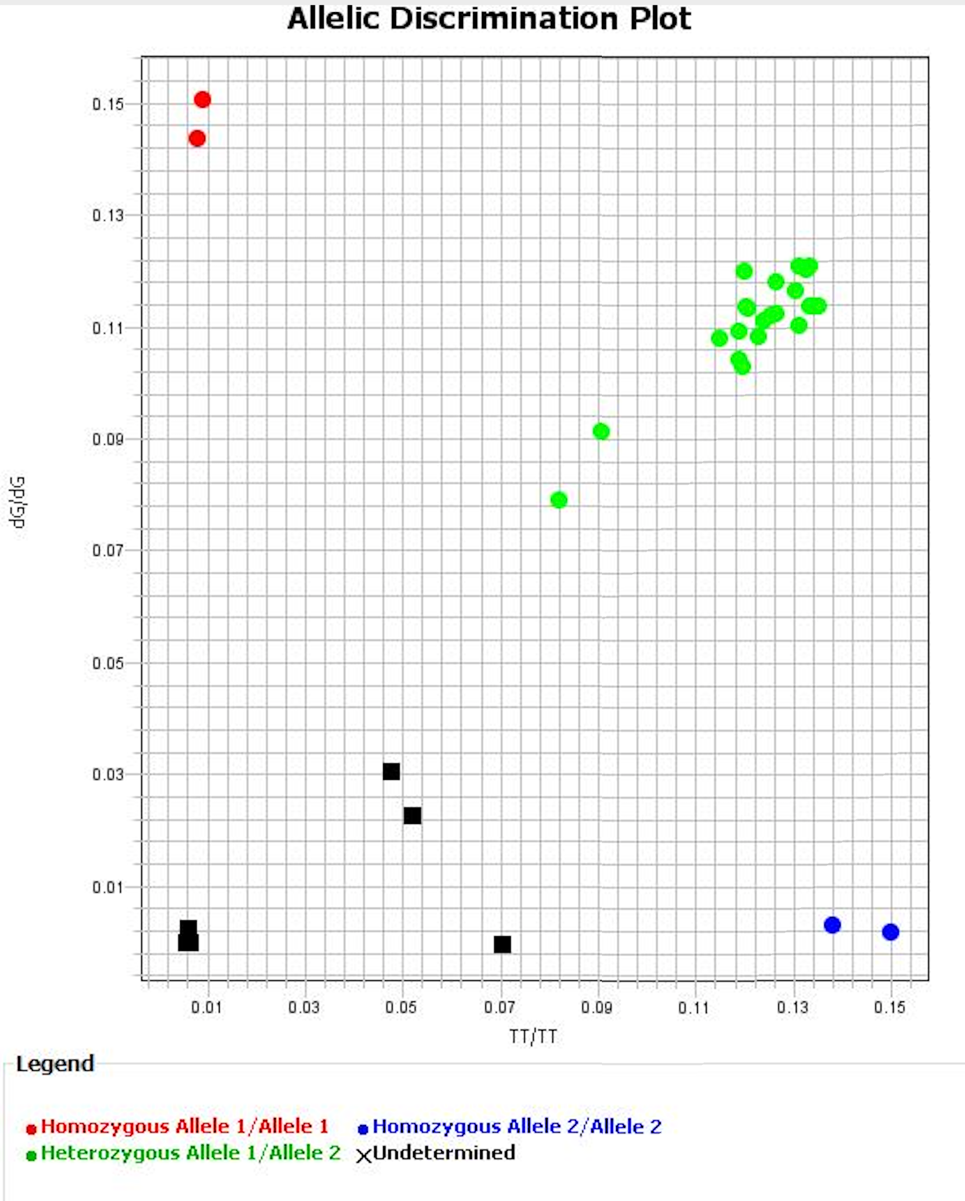


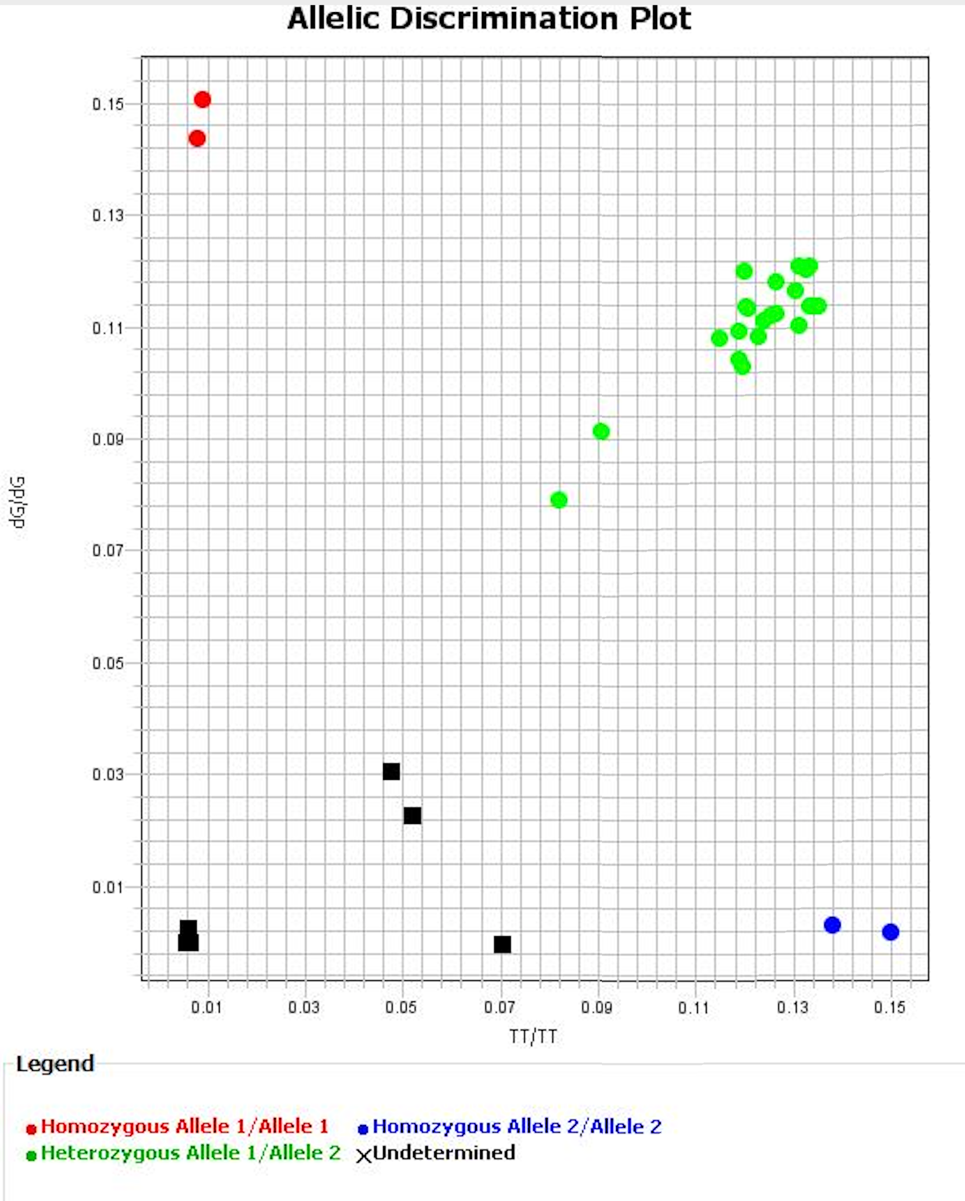


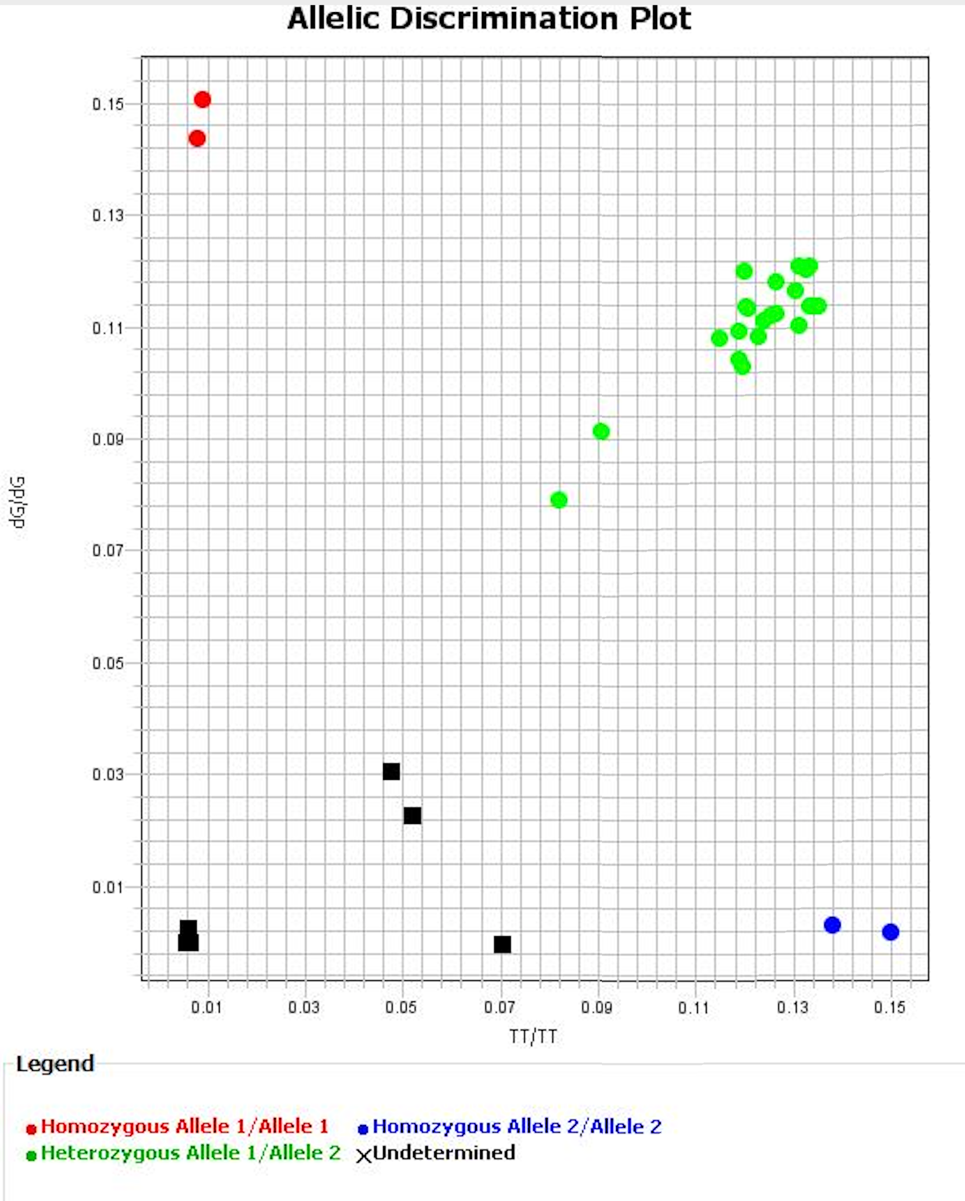


**dG/dG dG/TT**

**TT/TT Negative control**

Additional file 1: Fig. S1. Representative allelic discrimination plot for genotyping of IFNL4-rs368234815 polymorphism by custom TaqMan genotyping assay. A clear separation of the homozygotes is shown, heterozygotes on the other hand show presence of both alleles, with different expression levels between samples. HapMan controls are shown in duplicate, inside boxes, and five randomly selected study subjects gDNA samples are also included.
